# Microstructure Evolution of Rapid Solidified Invar Alloy

**DOI:** 10.3390/ma18030691

**Published:** 2025-02-05

**Authors:** Hanxin He, Zhirui Yao, Junfeng Xu, Xianzhe Shi, Xuyang Li

**Affiliations:** 1 School of Civil Engineering, Xi’an University of Architecture and Technology, No. 13 Yanta Road, Xi’an 710055, China; hehanxin@126.com; 2 School of Materials and Chemical Engineering, Xi’an Technological University, Xi’an 710021, China; yaozhiruiyx@163.com; 3 School of Materials Science and Engineering, Northwestern Polytechnical University, Xi’an 710072, China; 4 School of Aeronautics, Northwestern Polytechnical University, Xi’an 710072, China; shixianzhe@nwpu.edu.cn; 5 Xi’an Institute of Optics and Precision Mechanics, Xi’an 710119, China; lixuyang2004@126.com

**Keywords:** Invar alloy, EBSD, undercooling, grain boundary

## Abstract

Invar alloy has a wide range of applications in aerospace and precision instruments. However, the microstructure evolution during rapid solidification is not yet fully understood. In this study, the rapid solidification microstructure of Invar alloy with undercooling ranging from 5 K to 231 K was investigated using optical microscopy, EBSD, and TEM techniques. The results show that, as the undercooling increased from 5 K to 181 K, the microstructure transitioned from large dendrites to columnar grains and finally to small equiaxed grains. When the undercooling ranged from 181 K to 193 K, the grain size suddenly increased before continuing to decrease with further undercooling. EBSD analysis revealed that, for ΔT > 181 K, two distinct types of grains appeared in the microstructure: one larger and the other much smaller. Under low undercooling conditions, the grains grew anisotropically with a preferred orientation, while under high undercooling, there was no apparent preferred growth orientation. Many twin boundaries were observed in the high-undercooling samples, which were further confirmed by TEM analysis. Additionally, both twin boundaries and high-angle grain boundaries increased gradually with undercooling.

## 1. Introduction

Invar alloy, renowned for its ultra-low coefficient of thermal expansion, is a functional Fe–Ni alloy that finds wide applications in aerospace and precision instruments [1]. At present, there have been many research reports on Invar alloys. Silman studied the phase diagram and explained the “Invar” effect features of Fe-Ni alloys [2]. Acharya et al. investigated the ground state properties of Invar alloys via detailed study of the electronic structure of Fe_1−x_Ni_x_ alloys (x = 0.2, 0.3, 0.4, 0.5, 0.6, 0.7, 0.9) employing X-ray photoelectron spectroscopy and found that all the alloys exhibit soft ferromagnetic behavior with Curie temperature much higher than the room temperature, the results for Invar alloy Fe_0.6_Ni_0.4_ exhibit anomalous behavior [3]. Sagaradze studied the conditions for the formation of microconcentration inhomogeneities in Fe–Ni alloys [4]. Sui et al. studied the strengthening of the Fe-Ni Invar alloy through chromium, and they obtained the optimized result of a maximum strength of 644.4 MPa, together with a minimum coefficient of thermal expansion of 3.30 × 10^−6^ K^−1^ in the temperature range of 20–100 °C [5]. Si et al. studied the nanocrystalline Invar alloys prepared from Fe-Ni nanoparticles and found that the hardness can be significantly enhanced [6]. Dong et al. investigated the electro-plasticity of the Invar 36 alloy at different temperatures (100–600 °C) and found that under warm deformation, the flow stress decreases with the increase in temperature, which is directly attributed to dynamic recovery and dynamic recrystallization [7]. Zhu et al. studied the thermal expansion and mechanical properties of laser cladding of Invar alloy and found that the mechanical properties of the Invar alloy transverse cladding layer are better than those of the longitudinal cladding layer [8]. Huang et al. studied the additive manufacturing of Invar 36 alloy and summarized the microstructure mechanical properties of Invar alloy [9]. Singh et al. studied the elastic constants of equiatomic Fe–Ni Invar alloy single crystal and calculated Young’s modulus, shear modulus, bulk modulus, and Poisson’s ratio [10]. Schönfeld et al. studied the static atomic displacements in the near-surface region of the Invar alloy Fe-28 at. % Pt, and found that size-effect scattering is lower at room temperature than at 424 K [11]. Park et al. studied the microstructure-dependent etching behavior of a partially recrystallized Invar alloy and found that the overall etching behavior of the partially recrystallized Invar sheet not only depends on the properties of individual grains, such as orientation-dependent surface energy and geometrically necessary dislocation content but also on the properties of aggregates of grains such as grain boundary density [12]. Xu et al. studied the microstructure and mechanical properties’ modification of friction stir welded Invar alloy joint and developed an approach named large-load and low-speed friction stir [13]. Li et al. studied microstructure evolution and mechanical properties of laser metal deposition of Invar alloy and found that samples built by back-and-forth laser scanning pattern showed higher microhardness and tensile strength but lower elongation compared with samples built by one-way pattern, the horizontally built sample showed higher ultimate tensile strength and yield strength but lower elongation compared with the vertically built sample in the same laser scanning pattern [14].

Electron backscatter diffraction (EBSD) analysis can be used to study the microstructure orientation of alloy and the grain boundary change [15,16,17,18,19,20,21]. Hao et al. studied the microstructure evolution mechanism of copper single-phase alloys under deep undercooling conditions, and the significance of grain orientation and orientation difference distribution was found [15]. They also studied the nonequilibrium crystallization of highly undercooled Cu–Ni–Co alloys and found that the stress-induced recrystallization mechanism was the dominant factor in the grain refinement mechanism at large undercooling [16]. Wang et al. used EBSD technology to analyze the rapid solidification structure under high undercooling. They found the features of flat polygonal grain boundary, a high proportion of twin boundary, and a large proportion of high-angle grain boundary that confirmed the recrystallization of the solidified structure at high undercooling degrees [17]. Dai et al. studied the Fe_60_Co_20_Cu_20_ alloy rapidly solidified microstructures by EBSD technologies and found the α-Fe dendrites grow anisotropically with a preferred orientation on condition that undercooling is less than 178 K, and no apparent preferred growth orientation is found in the equiaxed grains once undercooling exceeds this critical value [18]. Lü et al. studied the orientation relationship and interface characteristics of the Ni_7_Zr_2_ and Ni_5_Zr phases in Ni-Zr hypoperitectic alloy by ESBD and found that the peritectic Ni_5_Zr phase is highly orientated, and its growth mode is almost parallel to the <110> directions [19]. Sahoo determined the orientation distribution of the Fe_50_Cu_50_ alloy microstructure from the aerodynamic levitation process by EBSD [21].

Recrystallization, which can be characterized by EBSD, generally occurs during the annealing process after a certain amount of plastic deformation of the material [22], such as cold-rolled Fe-34.5Mn-0.04C steel appears the partially recrystallized and fully recrystallized in annealing [23]. Xu et al. found that the recrystallization phenomenon also occurs in single-phase copper-based alloy samples solidified from high undercooling, which leads to grain refinement in solidification [24].

Although much has been studied on Invar alloys, there are few reports on the rapid solidification under deep undercooling conditions, so the mechanism and microstructure evolution of this alloy is still unclear. In order to reveal the microstructure evolution with solidification undercooling for Invar alloy, especially to show the microstructure forms in deep undercooling conditions, this study will adopt the glass fluxing technique to obtain the sample solidified from different undercooling. And then, optical microscopy, XRD, EBSD, and TEM technologies were used to analyze microstructure, the orientation distribution of grains rapidly solidified microstructures of Invar alloy. Finally, the twin boundaries in the microstructure are discussed.

## 2. Experimental

**Figure 1:**
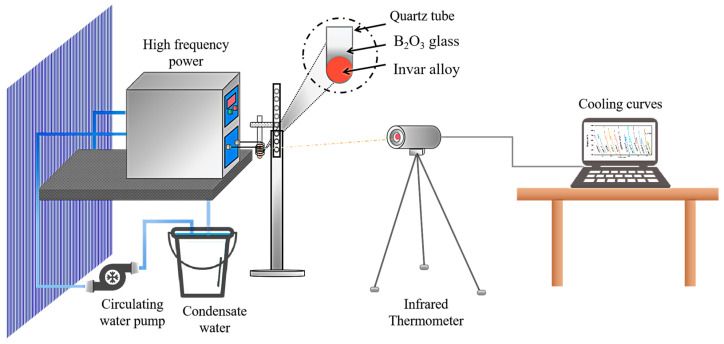
The schematic diagram for experimental techniques.

The master Invar alloy was made in Western Superconducting Technologies Co., Ltd. (Xi’an, China), and the composition has a content of ~36 wt.% Ni and ~63.8 wt.% Fe, the rest of the alloys are C, Si, P, Al, Zr, Fe, Cr, Mn, Co, S, Mg, and Ti. To investigate Invar alloy solidification in different undercooling conditions, the sample weighing about 4 g was put into a quartz crucible filled with B_2_O_3_ powder, then the crucible was placed in coils of a high-frequency induction melting furnace. The schematic diagram for experimental techniques is shown in Figure 1. The sample was cyclically heated and cooled until the desired undercoolings were achieved. The thermal curve of each sample was monitored by a one-color pyrometer with 10 ms delay time.

After solidification, the samples were cut and polished. Then, they were etched with a chemical solution (5 g FeCl_3_, 10 mL HCl, and 50 mL H_2_O) and then characterized by optical microscopy to observe the microstructures. The orientation relationships of microstructure were analyzed using an EBSD system installed in the SEM operated at 10 keV. For the preparation of EBSD test samples, Invar alloy samples solidified at different undercoolings were cut into thin slices with a thickness of 2 mm. First, use 1500 grit sandpaper for initial polishing and then use 3000 grit sandpaper for fine polishing and grinding. Then, electrolytic polishing is carried out in an electrolytic polishing solution at −10 °C, with a volume ratio of CH_3_COOH:HClO_4_ = 4:1, a current of 0.3–0.5 A, and a polishing time of 60 s. The equipment model used in the EBSD experiment is EDAX Hikari Plus (Western Superconducting Technologies Co., Ltd., Xi’an, China). Analyze the experimental results using OIM analysis software (https://www.edax.com/products/ebsd/oim-analysis, accessed on 18 January 2025). Twin grain analysis was performed on a transmission electron microscope (TEM, Thermo Fisher scientific TEM, Yanku Comprehensive Service Company, Shanghai, China, Tal0s F200S, where the electron beam acceleration voltage is 200 KV).

## 3. Results

### 3.1. Cooling Curves

**Figure 2:**
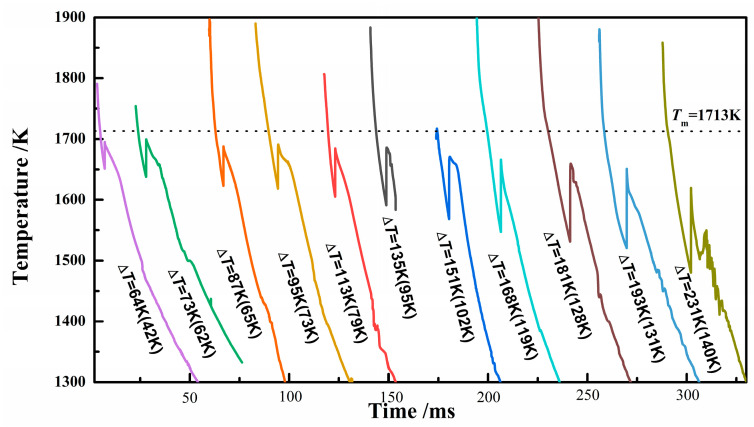
Cooling curves corresponding to various degrees of undercooling for Invar alloy.

The cooling curve is the first result from experiments, which can display the solidification undercooling and solidification rate. Figure 2 shows the cooling curves for the sample solidified with different undercoolings. Where undercooling Δ*T* (=*T*_m_ − *T*_N_, where *T*_m_ is the melting point of the alloy, *T*_N_ is the nucleation temperature) is the degree to which a liquid can be cooled below its melting temperature [25]. It can be seen that as the undercooling increased in Figure 2, the height of recalescence Δ*T*_R_ (=*T*_R_ − *T*_N_) increased, and the maximum temperature of recalescence (*T*_R_) decreased. This indicates that the degree of nonequilibrium solidification increased. Generally, the higher the undercooling, the faster the solidification rate, and the greater the supersaturation of the solidification structure. However, there is no exact theoretical formula for the relationship between the undercooling Δ*T* (=*T*_m_ − *T*_N_, *T*_N_ values require temperature calibration) and recalescence height Δ*T*_R_. Thus, we will list both the undercooling degree and recalescence height when describing a sample’s solidification condition.

### 3.2. Microstructures

**Figure 3:**
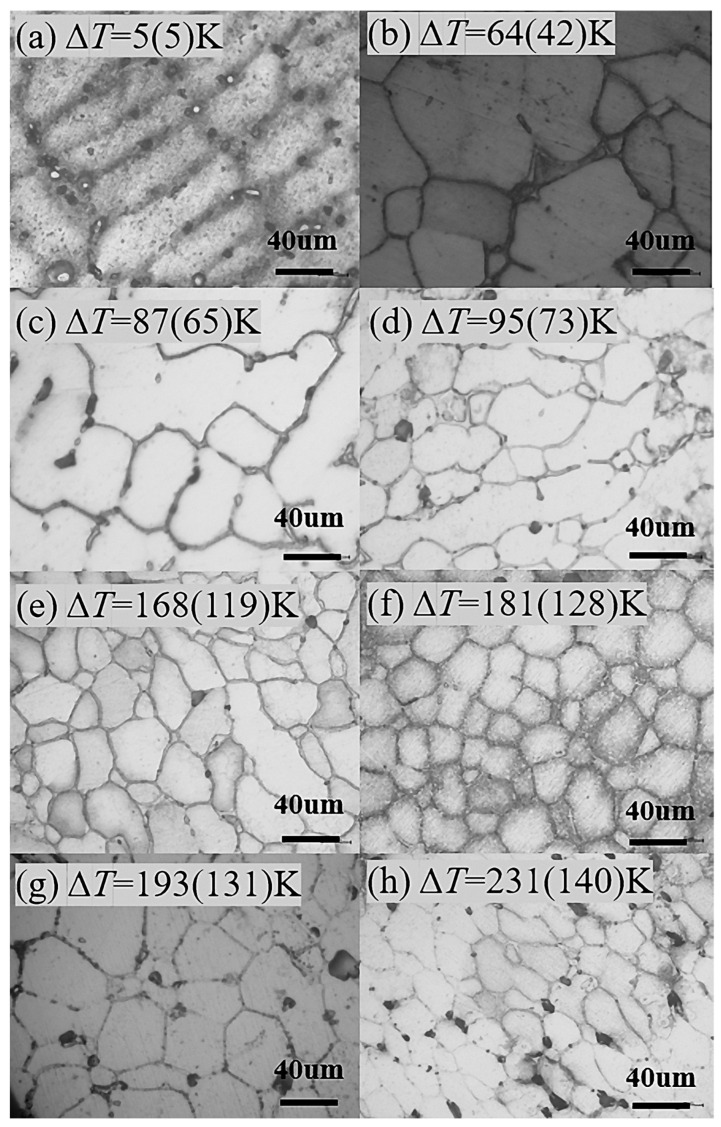
Microstructure from different undercooling degrees.

In general, the higher the solidification undercooling, the finer the microstructure for the alloy. In this study, the Invar alloy is no exception. The microstructures of Invar alloy solidified at different undercoolings are shown in Figure 3. It can be seen that when the undercooling is Δ*T* = 5 K (Δ*T*_R_ = 2 K), the microstructure exhibits a larger dendritic structure (Figure 3a). When the undercooling is Δ*T* = 64 K (Δ*T*_R_ = 42 K), the microstructure transforms into a mixed structure of dendritic and columnar crystals (Figure 3b). When the undercooling is Δ*T* = 87 K (Δ*T*_R_ = 65 K), the dendrites become finer, and the number of columnar crystals increases (Figure 3c). When the undercooling is Δ*T* = 95 K (Δ*T*_R_ = 73 K), the dendritic morphology almost disappears (Figure 3d). When the undercooling is Δ*T* = 168 K (Δ*T*_R_ = 119 K), the microstructure transforms into a mixed structure of columnar and equiaxed grains (Figure 3e). When the undercooling is Δ*T* = 181 K (128 K), the microstructure transforms into completely equiaxed grains, and the grain size is particularly fine (Figure 3f). When the undercooling reaches Δ*T* = 193 K (Δ*T*_R_ = 131 K), the sample microstructure is also completely equiaxed grains (Figure 3g), but it does not decrease in size. Instead, its size increases compared to Δ*T* = 181 K (128 K). When the solidification undercooling is Δ*T* = 231 K (140 K), the microstructure is equiaxed grains (Figure 3h), and the grain size of the microstructure decreases compared to Δ*T* = 193 K (Δ*T*_R_ = 131 K). It is worth noting that for the sample of the undercooling from Δ*T* = 181 K (Δ*T*_R_ = 128 K) to Δ*T* = 231 K (Δ*T*_R_ = 140 K), the grain size did not continue to decrease as expected. In addition, from Figure 3, the microstructure grains exhibit an internal arc from Δ*T* = 5 K (Δ*T*_R_ = 2 K) to Δ*T* = 168 K (Δ*T*_R_ = 119 K), or in other words, the centers of all grain boundaries point toward the interior of the grains. However, from Δ*T* = 181 K (Δ*T*_R_ = 128 K) to Δ*T =* 231 K (Δ*T*_R_ = 140 K), there are many grain interface centers pointing toward the outside of the grains. This suggests that some small grains may come from recrystallized structures. That is to say, when the undercooling is greater than 181 K (Δ*T*_R_ = 128 K), the microstructure undergoes recrystallization, causing the grain size of the microstructure to decrease further. Recrystallization is one way of grain refinement, but it generally occurs in samples that undergo plastic deformation [22]. Recrystallization occurs in samples solidified at extremely high undercooling, indicating that the sample from high undercooling has larger residual stresses. During the solidification process with large undercooling, the volume of the undercooled liquid phase will expand instantaneously when it solidifies, resulting in the system storing a certain amount of lattice distortion energy and retaining a certain amount of residual stress. In the subsequent cooling, the residual stress energy can be used as a recrystallization driver to support the recrystallization process, i.e., the release of lattice distortion energy promotes recovery and recrystallization, resulting in smaller grains.

**Figure 4:**
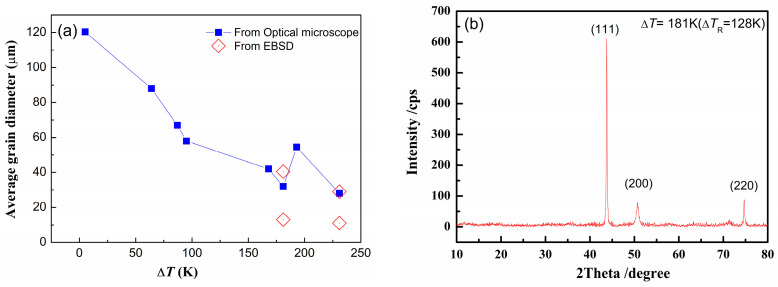
The grain size and the XRD pattern: (**a**) the relationship between average grain size and undercooling; (**b**) XRD result for the sample with Δ*T* = 181 K (Δ*T*_R_ = 128 K).

Figure 4a shows the average grain size as a function of undercooling from optical microscopy analysis results and EBSD results. It can be found that the larger the undercooling, the smaller the grain size if Δ*T* < 181 K (Δ*T*_R_ < 128 K). If Δ*T* > 181 K (Δ*T*_R_ < 128 K), from EBSD analysis, there are larger-size grains and smaller-size grains. In order to identify the crystalline structure, the sample with Δ*T* = 181 K (Δ*T*_R_ = 128 K) performed the XRD analysis, as shown in Figure 4b. It indicates that the sample has only one FCC (face-centered cubic) structure.

### 3.3. EBSD Analysis

To analyze the orientation differences and substructure of the samples, EBSD analysis was performed on the microstructures of samples solidified at higher undercooling degrees.

**Figure 5:**
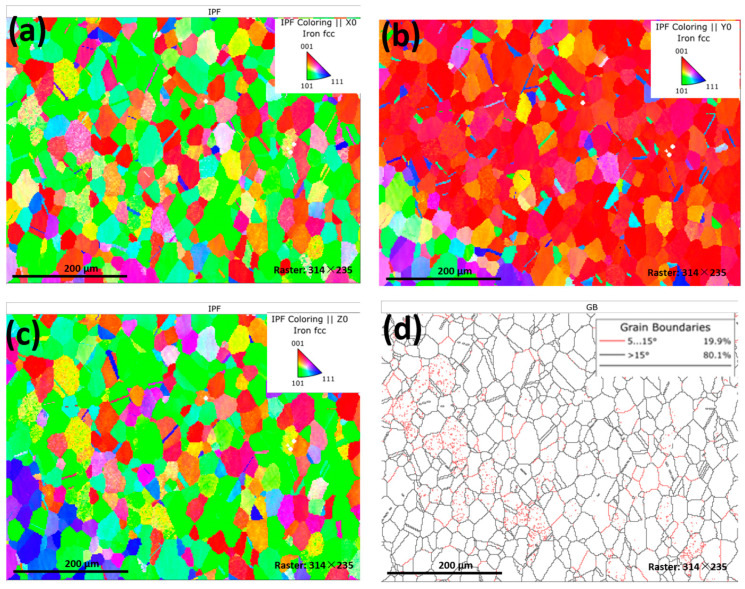
EBSD analysis of sample with Δ*T* = 181 K (Δ*T*_R_ = 128 K): (**a**) EBSD pattern of x direction; (**b**) EBSD pattern of y direction; (**c**) EBSD pattern of z direction; (**d**) low-angle and high-angle grain boundaries distribution.

Figure 5a–c show the EBSD analysis result of Δ*T* = 181 K (Δ*T*_R_ = 128 K) from three different axis directions of x, y, and z. It can be seen that the solidified grains are very small, and most equiaxed grains have significant randomness in orientation. The significant differences in orientation indicate that there may be many nuclei growing and forming equiaxed crystals in solidification. From the organizational photos, although there are grains in all directions, there are still more grains in the red [001] direction when viewed from the *y*-axis direction (Figure 5b), indicating that the sample grains still maintain a certain [001] texture orientation. Figure 5d shows the distribution of low-angle and high-angle grain boundary angles. The high-angle grain boundaries are marked in black, while the low-angle grain boundaries are marked in red. It can be seen from this that there are many low-angle grain boundaries distributed in the grains. Figure 5a–c also reveal the presence of some twin boundaries in the grains, indicating that under high undercooling conditions, not only small grains surrounded by high-angle grain boundaries can be obtained, but also subgrains enclosed by low-angle grain boundaries can be obtained. One reason for the small-size grain obtained is the dendrite fragmentation mechanism, i.e., the fragmentation of dendrites by remelting during the period following recalescence, where the interdendritic melt solidifies [26]. Another reason may be the occurrence of recrystallization [27,28]. One piece of evidence for recrystallization is the large number of twin boundaries, and another piece of evidence is that many subgrains can be observed. Due to the relatively short time for the recalescence process and latent heat release, the subgrains did not have enough time to grow up.

**Figure 6:**
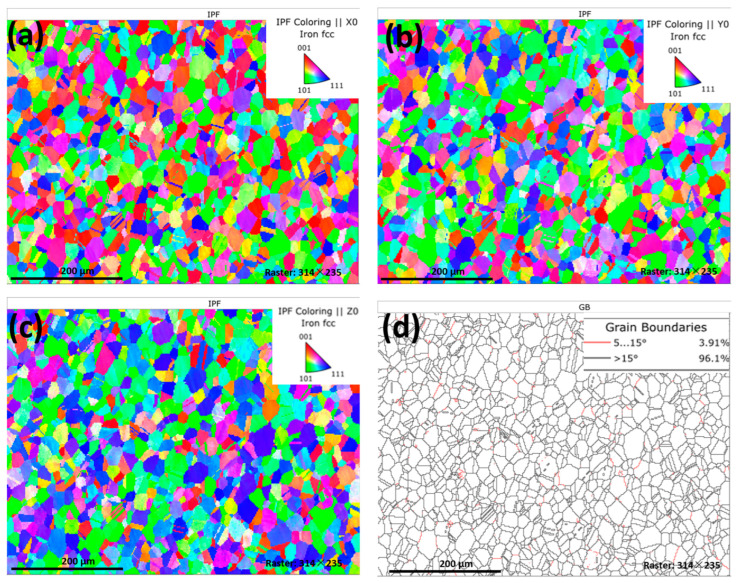
EBSD analysis of sample with Δ*T* = 231 K (Δ*T*_R_ = 140 K): (**a**) EBSD pattern of x direction; (**b**) EBSD pattern of y direction; (**c**) EBSD pattern of z direction; (**d**) low-angle and high-angle grain boundaries distribution.

The EBSD image for Δ*T* = 231 K (Δ*T*_R_ = 140 K) is shown in Figure 6, and it can be seen that the grains are significantly smaller than that of Δ*T* = 181 K (Δ*T*_R_ = 128 K). But from the optical microscope structure in Figure 3, at Δ*T* = 181 K (Δ*T*_R_ = 128 K), the microstructure actually becomes larger than that of Δ*T* = 64 K (Δ*T*_R_ = 42 K), indicating that some grain boundaries, such as twin boundaries and subgrain boundaries, cannot be observed in the metallographic structure of the optical microscope. However, EBSD grain orientation photos clearly show various grain boundaries. It can also be seen from Figure 6 that there are a large number of twin boundaries inside the sample with Δ*T* = 231 K (Δ*T*_R_ = 140 K), indicating that the recovery and recrystallization caused by the residual heat of recalescence are more obvious, and the number of twins is greater.

Comparing Figure 6a–c with Figure 5a–c, it can be observed that samples with higher undercooling not only have smaller grain sizes but also have more random grain orientations, indicating a higher nucleation rate.

Figure 5 and Figure 6 also indicate that the higher the solidification undercooling, the more twinning crystals will be formed in the sample due to recalescence heat. The higher the undercooling, the more growth orientations there are. That is, some obvious orientations can be seen during small undercooling solidification, and when solidification at large undercooling, the grain growth orientation becomes blurred.

**Figure 7:**
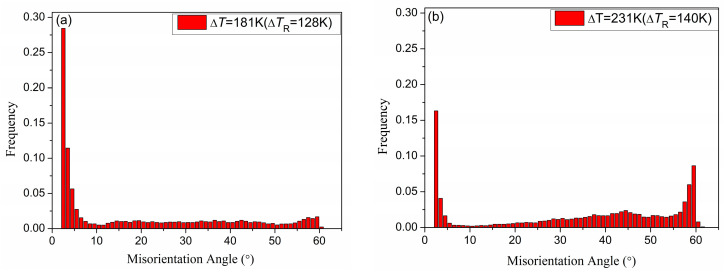
Misorientation angle distribution of the sample from different undercooling: (**a**) Δ*T* = 181 K (Δ*T*_R_ = 128 K); (**b**) Δ*T* = 231 K (Δ*T*_R_ = 140 K).

In order to study the variation in the angle between grains, Figure 7 presents the misorientation angle distribution of the samples solidified from Δ*T* = 181 K (Δ*T*_R_ = 128 K) to Δ*T* = 231 K (Δ*T*_R_ = 140 K). It shows that as the undercooling increases, the angle distribution gradually shifts toward the larger angle range of 55–60°. The main reason may be that the interface energy for the 60° angle of the grain arrangement of Invar alloy is the smallest, and the structure is the most stable. In addition, Figure 7 also illustrates that as the undercooling increases, the orientation angle between the grains of the alloy sample tends to be uniformly distributed, and the preference orientation is weakened.

Figure 5, Figure 6 and Figure 7 indicate that with increased solidification undercooling, the number of low-angle grain boundaries decreases while that of high-angle grain boundaries increases. There are two questions: (1) why so many low-angle grain boundaries appear in smaller undercooling samples; (2) why there are two size grains in large undercooling samples (Figure 6d).

For the first question, in a small undercooling sample, Karma considered it to be the fragmentation of dendrites by remelting during the period following recalescence [26]. When the large dendrites are just remelting but do not move enough distance, there appear many low-angle grain boundaries. Xu et al. considered that recrystallization occurs in the recalescence process [24]. The low-angle grain boundaries indicate the state was in the recovery stage, and recrystallization does not start in smaller undercooling.

For the second question, as shown in Figure 6d, there are many small-size grains and some large-size grains, indicating incomplete recrystallization, while the number of subgrains is significantly very small. The large-size grains are without recrystallization, and the smaller grains are from completed recrystallization. In principle, when recrystallization occurs, the time has already exceeded the recovery stage, and the subgrain boundaries will disappear at the end of recovery. In the recrystallization stage, all residual stresses must be released, and the subgrain will completely disappear at the end of crystallization. That is to say, the subgrain boundaries must also move with the grain boundaries, either disappearing or merging into the high-angle grain boundaries. As shown in Figure 5d and Figure 6d, there are very few residual low-angle grain boundaries, indicating that most of the areas have completed the recovery and transformation and have undergone recrystallization. Therefore, the Invar alloy undergoes recrystallization during the recalescence stage under high undercooling solidification conditions. That is to say, the grain refinement of the undercooled solidified samples is mainly attributed to the occurrence of recrystallization in large solidification undercooling, but the source of this driving force still needs to be discussed in the following.

**Figure 8:**
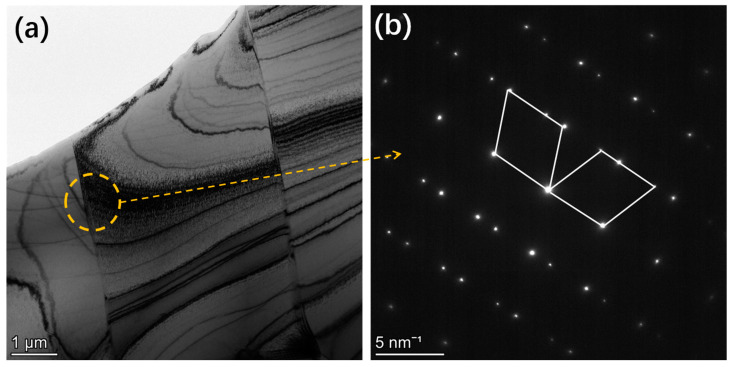
TEM analysis of the sample from undercooling of ΔT = 181 K (ΔT_R_ = 128 K): (**a**) the microstructure; (**b**) the diffraction patterns of twins.

In order to identify the grain twin of the sample solidified from high undercooling, the sample with Δ*T* = 181 K (Δ*T*_R_ = 128 K) was analyzed by TEM, as shown in Figure 8. It can be found that the grain twins have a large size (Figure 8a). Figure 8b shows the diffraction patterns of twins. One is bright, and the other is dark. This result confirms that the twin structure appears in the sample solidified from large undercooling.

## 4. Discussion

As mentioned above, in the case of particularly high undercooling solidification, the refinement of the solidification structure is related to the mechanism of recovery and recrystallization. The driving force for recrystallization is not the Gibbs free energy difference of the system but the lattice distortion energy of the material. In the case of extremely high undercooling during solidification, how does lattice distortion energy occur? We consider that under extremely high undercooling, the density and viscosity of the undercooled liquid phase of the Invar alloy increase. Subsequently, many nuclei grow simultaneously. Then, the volume expansion from the liquid phase to the solid phase would occur, but due to the high solidification rate and incomplete volume expansion process, atoms are frozen in a nonequilibrium state, resulting in many lattice distortions. As a result, a large amount of lattice distortion energy is stored inside the system during the solidification with high undercooling. As the solidification interface continues to move forward, the latent heat released by the solidification interface gradually transfers to the already solidified region. This is equivalent to annealing the already solidified region, providing heat for the start of recrystallization. Recovery and recrystallization occurred. However, due to the extremely limited latent heat energy of crystallization, it is quickly consumed, so recovery and recrystallization cannot be completely carried out. Thus, some subgrain boundaries can still be seen in the final solidified sample (Figure 5d and Figure 6d), and many small grains can also be seen at the same time, which are the products of recrystallization. Therefore, it is speculated that the driving force for the occurrence of recrystallization in extremely undercooled solidification comes from the lattice distortion energy caused by insufficient volume expansion before and after solidification.

From Figure 3, the grain size in Δ*T* = 193 K (Δ*T*_R_ = 131 K) appears as larger than Δ*T* = 181 K (Δ*T*_R_ = 128 K). Karma studied the dendrite growth behavior and microstructural changes as a function of undercooling for Ni_70_Cu_30_ alloy and found the existence of two consecutive microstructural transitions with increasing undercooling. As shown in Figure 1 in Ref. [26], a grain-refined equiaxed microstructure is observed at smaller undercoolings (Δ*T* < Δ*T*_1_*), a coarse-grained dendritic morphology at intermediate undercoolings (Δ*T*_1_* < Δ*T* < Δ*T*_2_*), and a reentrance of the grain-refined equiaxed microstructure at larger undercoolings (Δ*T* > Δ*T*_2_*). Karma considered it to be the fragmentation of dendrites by remelting during the period following recalescence, where the interdendritic melt solidifies, i.e., the refined grains are from dendrite fragmentation. Xu et al. consider grain refined from recrystallization occurred at large undercooling [27,28]. But, they did not notice that there were actually two types of equiaxed grains in the microstructure obtained under high undercooling solidification conditions. One is larger in size, the other is smaller in size, and the two coexist. The larger-size grain may be the result of dendrite fragmentation by the release of latent heat of crystallization during solidification. And the smaller size grain is distributed in the larger grain, which, with high lattice distortion energy, is the result of recrystallization. All average grain sizes always decrease with increasing undercooling. However, after solidification under high undercooling conditions, some grains remain as subgrain boundaries, which were not easily discovered by optical microscopy in the past.

## 5. Conclusions

The microstructure evolution of Invar alloy during rapid solidification was investigated using optical microscopy, XRD, EBSD, and TEM techniques. The results show that, as undercooling increases, the microstructure transitions from large dendrites to columnar grains and eventually to fine equiaxed grains. At low undercooling, the grains grow anisotropically with a preferred orientation, while at higher undercooling, the grains exhibit no clear preferred growth direction. Additionally, a significant number of twin boundaries appeared in the samples under high undercooling, as confirmed by TEM analysis. The formation of twin grains increases with higher solidification undercooling. The refinement of the microstructure can be attributed to dendrite fragmentation and recrystallization mechanisms. This study provides valuable insights into the microstructure formation mechanisms of Invar alloys.

## Data Availability

The original contributions presented in this study are included in the article. Further inquiries can be directed to the corresponding author.
